# Mutations in Arg143 and Lys192 of the Human Mast Cell Chymase Markedly Affect the Activity of Five Potent Human Chymase Inhibitors

**DOI:** 10.1371/journal.pone.0065988

**Published:** 2013-06-19

**Authors:** Parvin Ahooghalandari, Nina Hanke, Michael Thorpe, Andreas Witte, Josef Messinger, Lars Hellman

**Affiliations:** 1 Department of Cell and Molecular Biology, Uppsala University, The Biomedical Center, Uppsala, Sweden; 2 Abbott Products, Hannover, Germany; University of Delhi, India

## Abstract

Chymotrypsin-like serine proteases are found in high abundance in mast cell granules. By site-directed mutatgenesis, we have previously shown that basic amino acids in positions 143 and 192 (Arg and Lys respectively) of the human mast cell chymase are responsible for an acidic amino acid residue preference in the P2' position of substrates. In order to study the influence of these two residues in determining the specificity of chymase inhibitors, we have synthesized five different potent inhibitors of the human chymase. The inhibitory effects of these compounds were tested against the wild-type enzyme, against two single mutants Arg143Gln and Lys192Met and against a double mutant, Arg143Gln+Lys192Met. We observed a markedly reduced activity of all five inhibitors with the double mutant, indicating that these two basic residues are involved in conferring the specificity of these inhibitors. The single mutants showed an intermediate phenotype, with the strongest effect on the inhibitor by the mutation in Lys192. The Lys192 and the double mutations also affected the rate of cleavage of angiotensin I but did not seem to affect the specificity in the cleavage of the Tyr_4_-Ile_5_ bond. A more detailed knowledge about which amino acids that confer the specificity of an enzyme can prove to be of major importance for development of highly specific inhibitors for the human chymase and other medically important enzymes.

## Introduction

Mast cells (MC) are distributed along both external and internal surfaces of the body. They are resident tissue cells that are frequently found in the connective tissue of the skin and around blood vessels and nerves [1]. Mucosal MC, another subtype of MC, are also found in the mucosa of the airways and the intestine. Due to their tissue location MC are among the first cells to encounter bacteria, viruses and other foreign material that enter our tissues [1]. These cells store a large amount of potent mediators in their cytoplasmic granules and the majority of the proteins found within these granules are serine proteases [2], [3], [4], [5]. These abundant granule proteases are stored in tight complexes with negatively charged proteoglycans and are released into the extracellular environment in response to immunological and neuronal stimuli [6], [7]. One subfamily of these proteases is the chymotrypsin-like chymases, which cleave at the C-terminal side of aromatic amino acids (aa) in substrates. Phylogenetic analyses of the chymases have identified two distinct subfamilies, the α-chymases and the β-chymases [8], [9], [10], [11]. The α-chymases are found as a single gene in all species investigated, except for ruminants where two very similar α-chymase genes have been identified [12], whereas functional β-chymases have only been identified in rodents.

We have previously determined the cleavage specificity from position P4 to P3' in the human chymase (HC) [13]. Besides the primary specificity for P1 Phe or Tyr, the strongest preference observed was for negatively charged (acidic) aa residues in the P2' position. Many natural substrates for the HC also hold acidic aa residues in the P2' position [13], [14]. These observations suggested an important role for negatively charged aa in the P2' position during substrate discrimination by the HC. The structure of the HC has been extensively investigated, which has provided insight into important enzyme/substrate interactions. These studies have shown that Lys40, Arg143 and Lys192 are located close to the S2' binding site, which may favour negatively charged P2' side chains of substrates. In a recent report, we have tested the role of Arg143 and Lys192 as P2' specificity determining residues. By *in vitro* mutagenesis the HC coding region was modified to substitute Arg143 for Gln and Lys192 for Met (plus a double mutant containing both mutations), which are residues found in the same positions of chymases that lack acidic P2' specificity [15]. Our results clearly showed that positions 143 and 192 mediate the acidic P2' specificity. This finding made us interested to see whether various chymase inhibitors under clinical development depend on these two residues for their specificity towards the HC. In order to address this question, we have synthesized five potent HC inhibitors originating from five different companies. The compounds we have tested are: A, a compound, TY51184, from Tao Eiyo (patent no WO 2002 022595), B, a compound from Teijin (patent no WO 2007 068621), C, a compound from Johnson & Johnson (patent no WO 2005 073214), D, a compound from Roche (patent no WO 2000 003997) and E, a compound from Boehringer Ingelheim (patent no WO 2009 023655). The potency of the inhibitors is similar in wild-type (wt) HC towards the chromogenic substrate L-2130 (Suc-Leu-Leu-Val-Tyr-pNA, Bachem, Bubendorf, Switzerland) with pIC50 values of 7.5, 7.6, 7.8, 7.8 and 7.3 uM, respectively. These compounds were tested against four different HC variants, the wt, the Arg143Gln and Lys192Met single mutants and the double mutant, focusing on their activity to inhibit cleavage of a known synthetic chymase substrate. Our results show that all five inhibitors are dependent on both the Arg143 and Lys192 for their specificity to the enzyme. However, the most prominent effect was observed by mutation of the Lys in position 192. We have also studied the role of these two aa in conferring specificity to the cleavage of angiotensin I (Ang I) to Ang II. Our results indicate that the Lys192 mutation is of importance for the rate of Ang I cleavage but does not seem to affect the specificity towards the cleavage of the Tyr_4_-Ile_5_ bond. These findings are further supported by information obtained from X-ray structures of compound C (PDB: 2HVX) and an analogue of compound B (PDB: 3SON) from Boehringer (http://www.rcsb.org/pdb/home/home.do; RSCB Protein Data Bank) [15]. Detailed information concerning which residues are responsible for the specificity of the enzyme could potentially be of major importance in foreseeing the specificity of an effective inhibitor. Such information may prove very valuable in the efforts to develop highly specific inhibitors for the clinic.

## Materials and Methods

### In vitro mutagenesis of the human chymase

The construction of the expression plasmids for both the HC wt enzyme and the mutants has previously been described in detail [13], [15].

### Production and purification of recombinant human chymase mutants

The vector constructs encoding the mutants of the HC were transfected into the human embryonic kidney cell line (HEK 293 EBNA (Invitrogen, Carlsbad, CA, USA)) at approximately 80% confluency, using Lipofectamine (Invitrogen) as previously described [16]. Selection of transfected cells was initiated by the addition of 1.5 µg/ml puromycin to the cell culture medium (DMEM supplemented with 5% FCS, 50 µg/ml gentamicin and 5 µg/ml heparin). The level of puromycin was decreased to 0.5 µg/ml after approximately seven days of selection.

Conditioned medium was collected, filtered and centrifuged to remove cell debris, followed by the addition of 300 µl (nickel-nitrilotriacetic acid) Ni-NTA agarose beads (Qiagen, GmbH, Hilden, Germany) per litre of conditioned medium. After 1 h of incubation with gentle agitation at 4°C, the beads were pelleted by centrifugation and transferred to 1.5 ml reaction tubes (Trefflab, Degersheim, Switzerland). The collected Ni-NTA beads were then transferred to a 2 ml column and washed five times with washing buffer (1 M NaCl, 0.05% Tween in PBS). Bound protein was then eluted with elution buffer (100 mM imidazole, 0.2% TritonX-100 in PBS). Protein purity and concentration was estimated by separation on 12.5% SDS-PAGE gels (Invitrogen,). Protein samples were mixed with sample buffer, and β-mercapto-ethanol was added to a final concentration of 5%. To visualize the protein bands, the gel was stained with colloidal Coomassie Brilliant Blue according to previously described procedures [17].

### Activation of recombinant human chymase variants

Approximately 30 µg of each HC mutant was diluted 1∶2 in ddH_2_O and digested for 5 h at 37°C with EKMax™ enterokinase (Roche, Germany), using one unit per 10 µg of recombinant protease.

Enzymatic activity was measured towards the chromogenic substrate L-2130 (Suc-Leu-Leu-Val-Tyr-pNA, Bachem, Bubendorf, Switzerland). Measurements were performed in 96-well microtiter plates with a substrate concentration of 0.18 mM in 200 µl PBS. L-2130 hydrolysis was monitored spectrophotometrically at 405 nm in a Versamax microplate reader (Molecular Devices, Sunnyvale, CA).

### Inhibitor assay

Enzymatic activity during the inhibitor assay was measured towards the chromogenic substrate L-2130 (Suc-Leu-Leu-Val-Tyr-pNA) (Bachem, Bubendorf, Switzerland). Measurements were performed in 96-well microtiter plates with a substrate concentration of 0.2 mM in 200 µl PBS. L-2130 hydrolysis was monitored spectrophotometrically at 405 nm in a Versamax microplate reader (Molecular Devices, Sunnyvale, CA). The reaction was performed in 200 µl total volume consisting of 145 µl of PBS, 5 µl of the substrate solution (8.0 mM L-2130 substrate in DMSO) and 50 µl enzyme+inhibitor in PBS (approximately 0.5 ug of the enzyme in PBS for the 200 µl reaction). The final concentration of DMSO was approximately 2.5% in the reaction. The inhibitor concentration was 0.6 µM for inhibitors A, B, D and E and 0.2 µM for inhibitor C. The experiments were performed four times with very similar results.

### Analysis of the cleavage products of Ang I, after cleavage with human chymase and chymase mutants

Ten µg of 95–98% pure Ang I (Sigma-Aldrich, Stockholm, Sweden) was digested with 17 ng of the HC, double mutant, Arg143 or Lys192 single mutants for 1 hour at 37°C in a PBS buffer. The cleavage products were then analysed by mass spectrometry (MS). The experiments were performed three times and the results from the three experiments were almost identical.

## Results

### Production and purification of four recombinant human chymase variants

**Figure 1:**
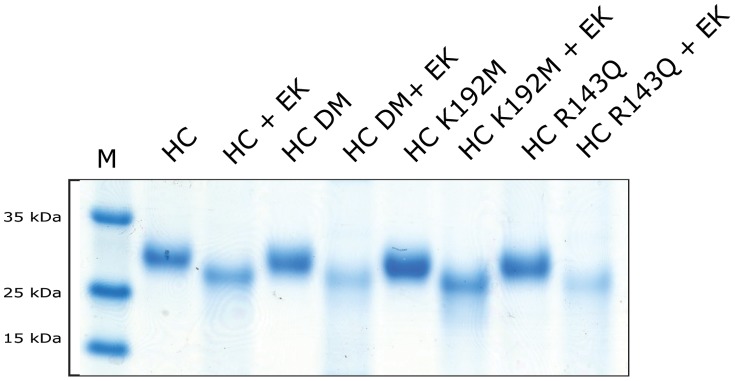
Purification and activation of recombinant HC and three mutants. The figure shows an SDS-PAGE analysis of inactive and active enzymes used in the inhibitor assay. The wt enzyme and three different recombinant HC mutants were expressed with an N-terminal His_6_-tag followed by an EK susceptible sequence replacing the signal peptide. These pro-enzymes were first purified on Ni-NTA beads (−EK) and then activated by removal of the His_6_-tag by EK digestion (+EK). Following activation, the enzymes were analyzed by separation on SDS-PAGE and visualized with Coomassie Brilliant Blue staining.

Two single mutants of the HC; Arg143Gln and Lys192Met, and a double mutant, Arg143Gln+Lys192Met, have previously been produced by *in vitro* mutagenesis [15]. The coding regions for the active protease of these three mutants and the wt enzyme were inserted into the mammalian expression vector pCEP-Pu2 [16]. To facilitate the purification and activation of the recombinant protease, the coding region of the active protease was preceded by a region encoding a six histidine (His_6_) tag and an enterokinase (EK) site (Fig. 1 A). Following transfection into HEK 293 EBNA cells, recombinant protein was purified from conditioned media on Ni-NTA agarose, by binding through the N-terminal His_6_-tag. The protein yield was between 100–150 µg recombinant protein from one litre of medium for all variants of the enzyme.

### Activation of recombinant human chymase mutants

Following the Ni-NTA agarose purification the enzymes were activated by removal of the His_6_-tag by proteolytic cleavage with EK. Approximately 30 µg of each enzyme were subjected to EK for 5 h at 37°C. Samples of inactive and activated proteases were separated on SDS-PAGE gels in order to ensure successful removal of the His_6_-tag and the EK susceptible cleavage site (Fig. 1B). Like the wt enzyme, the mutated inactive proteases migrated as 27 kDa bands and the EK digested, active enzymes as 26 kDa bands (Fig. 1B). The purified proteins were analysed on SDS-PAGE gels to determine purity and concentration of the recombinant enzymes (Fig. 1B).

The proteolytic activity of the eluted fractions of the three mutated HC proteins were analyzed by cleavage of the chymotrypsin sensitive chromogenic substrate L-2130 (Suc-Leu-Leu-Val-Tyr-pNA, Bachem, Bubendorf, Switzerland).

### Synthesis of five human chymase inhibitors

**Figure 2:**
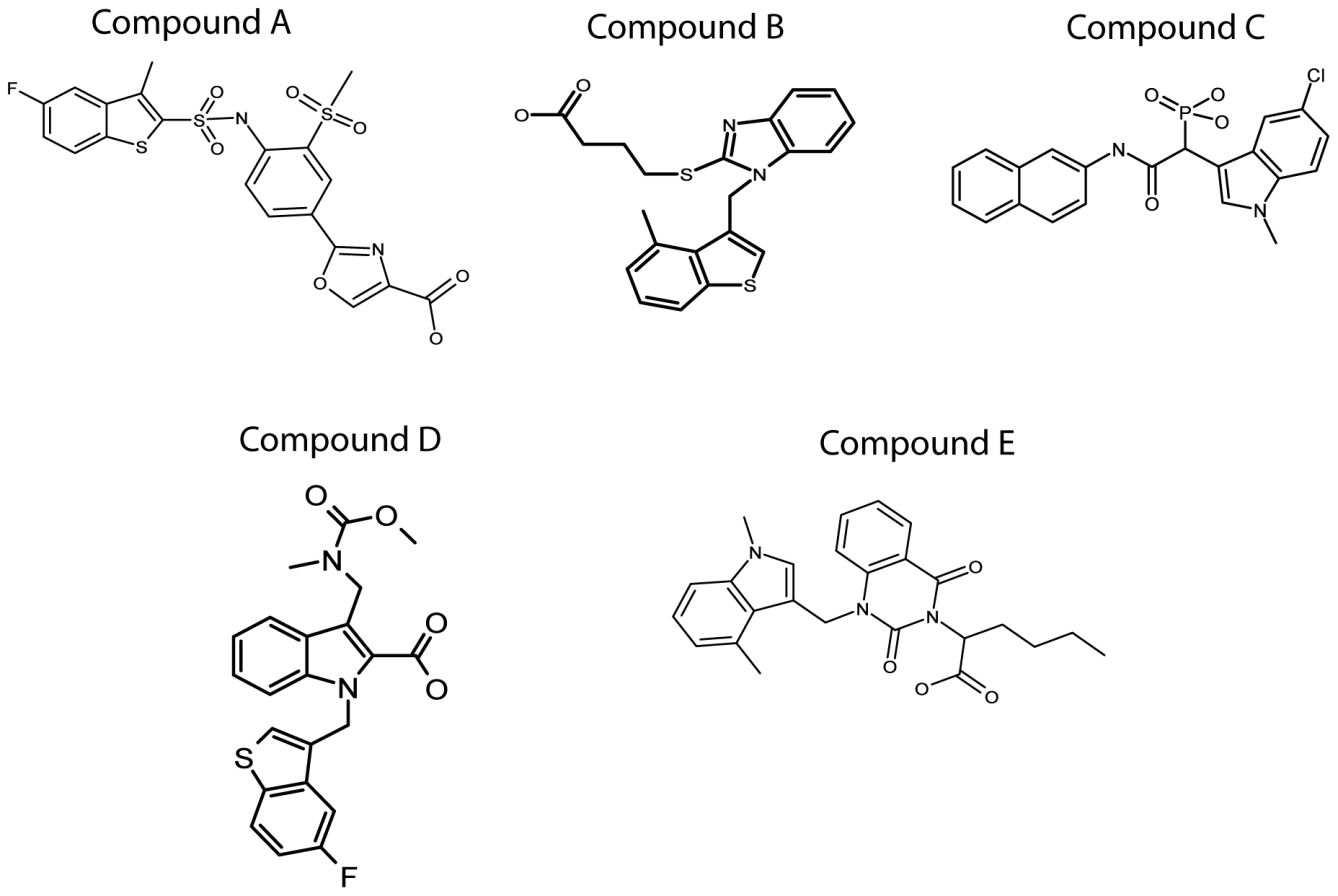
Structures of the five different chymase inhibitors used in this study. Five different patented inhibitors of the HC originating from 5 different companies were synthesized. The inhibitors were subsequently used to determine the dependence of the basic amino acids Arg143 and Lys192 for the specificity in the interaction between the enzyme and the inhibitor. Compound A (TY51184) originates from Tao Eiyo (patent no WO 2002 022595), compound B from Teijin (patent no WO 2007 068621), compound C from Johnson & Johnson (patent no WO 2005 073214), compound D from Roche (patent no WO 2000 003997) and compound E from Boehringer Ingelheim (patent no WO 2009 023655).

Five different HC inhibitors were synthesized by conventional chemical synthesis, to use as reference compounds for the development of novel chymase inhibitors and for mechanistic studies. The compounds tested were: A, TY51184 a compound from Tao Eiyo (patent no WO 2002 022595), B, a compound from Teijin (patent no WO 2007 068621), C, a compound from Johnson & Johnson (patent no WO 2005 073214), D, a compound from Roche (patent no WO 2000 003997) and E, a compound from Boehringer Ingelheim (patent no WO 2009 023655). A schematic drawing of the structures of these five inhibitors is presented in figure 2. All five inhibitors show clear similarities in structure, with two or more aromatic ring structures. One of these aromatic rings probably directly interacts with the binding pocket in the active site of the HC. The crystal structure of the HC with one of these bound inhibitors is available, which provides interesting clues about the role of Arg143 and Lys192 for the interaction between the protease and inhibitor. The structural requirements for these interactions will be discussed in more detail in the coming sections.

### Determining the influence of Arg143 and Lys192 on the interaction between five different human chymase inhibitors and the human chymase

**Figure 3:**
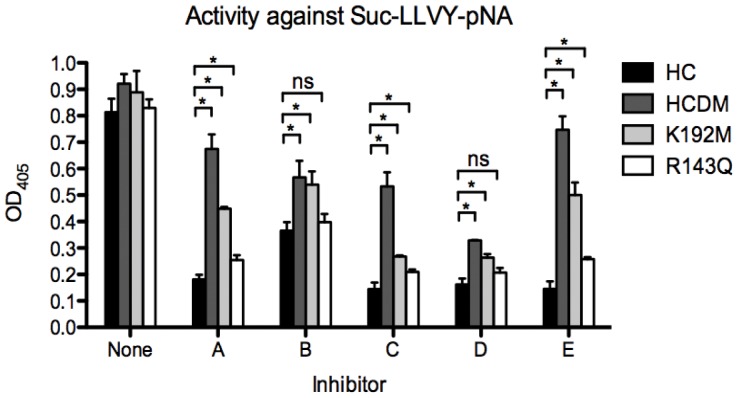
Analysis of the inhibition by five different HC inhibitors on the cleavage of a recombinant substrate by the wt HC and three mutants. The activity of five inhibitors were analysed in a 200 µl reaction volume using approximately 0.5 µg of the purified enzyme and 0.18 mM of the synthetic substrate L-2130 (Suc-Leu-Leu-Val-Tyr-pNA). The inhibitor concentration was 0.6 µM for inhibitors A, B, D and E and 0.2 µM for inhibitor C. The reaction was first normalized without inhibitor so that the amount of chymase should give approximately the same amount of substrate cleavage before analysing the effect of the inhibitor (first panel). The experiments were performed four times (fully independently) with almost identical results. The standard deviations between the four experiments (means ± SD) are included in the figure. Statistical analyses were performed using the Mann-Whitney test with two-tailed P value and 95% confidence interval parameters. All P values = 0.0286, *P<0.05, ns, not significant.

In order to study the role of the two basic amino acids Arg 143 and Lys 192 in determining the specificity of the interaction between the inhibitor and the enzyme, the HC and mutants were analyzed by using the chymotrypsin sensitive chromogenic substrate L-2130 (Suc-Leu-Leu-Val-Tyr-pNA, Bachem, Bubendorf, Switzerland) in the presence of a carefully titrated amount of each inhibitor. We used a synthetic substrate lacking amino acids C-terminal of the cleavage site to minimize the effect of the mutants on the cleavage of the substrate. This should give a more unbiased reading when assessing the effect of the inhibitors. The concentration of the protein was first titrated against the synthetic substrate in the absence of an inhibitor. The same concentration of the enzyme was then used in the presence of the different inhibitors. The inhibitor concentrations were also carefully titrated to provide between 50–80% inhibition of the wt enzyme, in order to have maximal resolution in the analysis. The optimal inhibitor concentration was found to be 0.6 µM for all inhibitors (A, B, D and E) except C, where the optimal concentration was 0.2 µM. The experiments were independently performed four times with almost identical results (Fig. 3).

The analysis showed that the double mutant displayed a markedly reduced sensitivity towards all five inhibitors (Fig. 3). The most pronounced effect of these two mutations was seen with inhibitors A, C and E (Fig. 3). A cooperative effect of the two mutations was seen for all five inhibitors. However, when comparing the effect of the two single mutants, the mutation of Lys192 was found to have the most prominent effect on the inhibitor activity (Fig. 3). For one of the inhibitors, compound B, almost all of the effect was seen with the Lys192 mutation and only a very minor additive effect was observed with the Arg142 mutation (Fig. 3).

### The role of Arg143 and Lys192 for the specificity in cleavage of angiotensin I by HC

**Figure 4:**
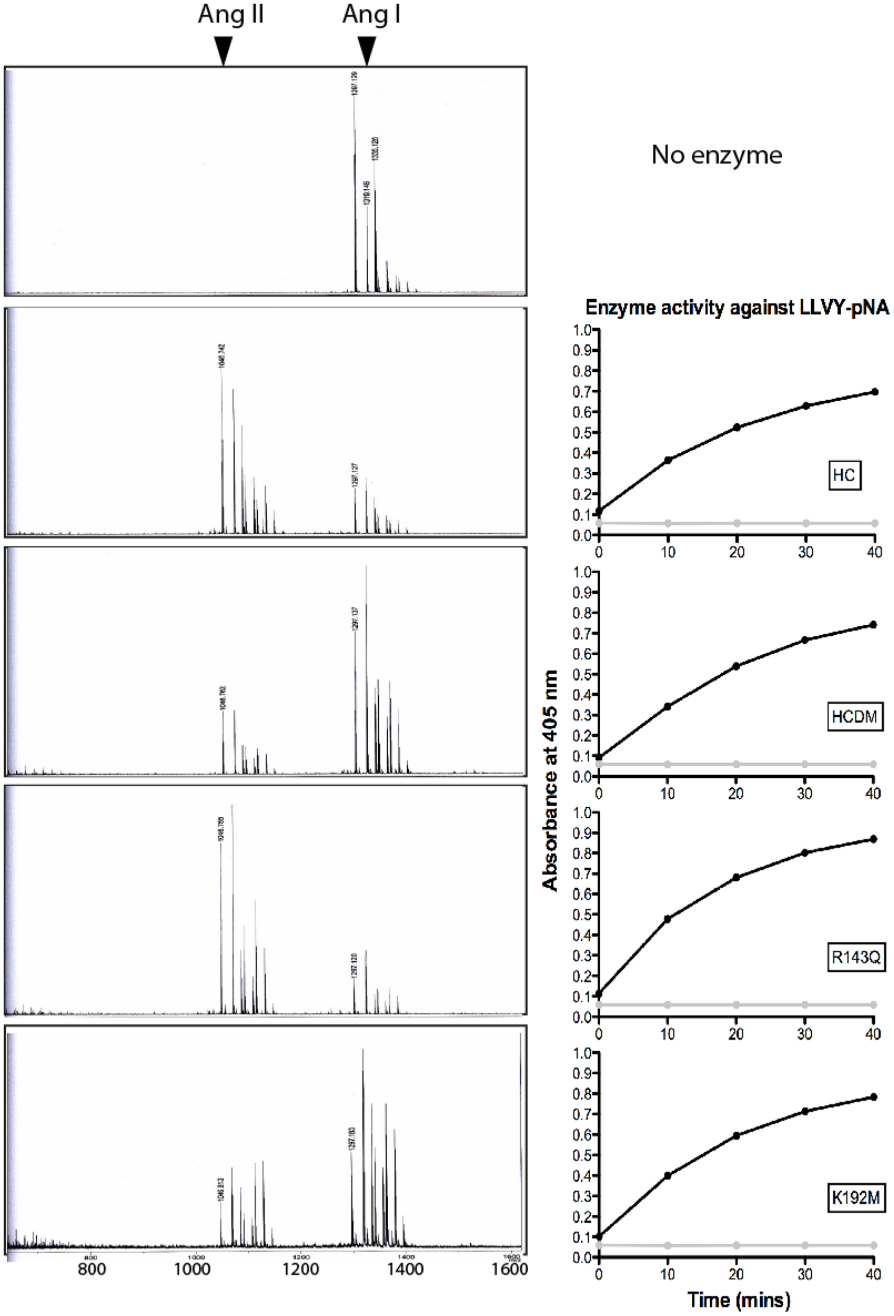
The effect of mutations in Arg143 and Lys192 mutants on the processing of Ang I. The Arg143 and Lys192 variants ability to process Ang I was analysed by cleaving 10 µg of Ang I in a 20 µl reaction volume. Ang I (Asp_1_-Arg_2_-Val_3_-Tyr_4_-Ile_5_-His_6_-Pro_7_-Phe_8_-His_9_-Leu_10_, MW∼1297) is converted to Ang II (MW∼1046) by cleavage after the Phe_8_ bond. As reference samples we also used the wt enzyme and the double mutant (Arg143+Lys192). Approximately the same amount of protease, as determined by cleavage of the chromogenic substrate LLVY-pNA, was used in each reaction (Right panel). Two µl samples were removed at different time points and analysed by mass spectrometry. The obtained results were compared in order to estimate the generated products aswell as a semi-quantitative measurement of the cleavage rate and its dependence on residues Arg143 and Lys192 and the double mutant. These experiments were independently performed three times with similar results.

Ang I has been the focus of many chymase studies and with particular attention to the roles of Lys40 and Arg143 in the interaction with Ang I. However, the role of Lys192 has never been addressed in detail. We therefore decided to use 10 µg of Ang I with the four different HC variants and to analyze the cleavage products by mass spectrometry. The results from this analysis showed a diminished effect on the cleavage rate with the double mutant. However, no or only a minor effect was observed on the specificity (data not shown). No cleavage at the Ang I degrading Tyr4-Ile5 bond was seen with the double mutant (data not shown)(Fig. 4). The decreased effect on the cleavage rate was almost exclusively seen with the Lys192 mutation as the Arg143 mutation had no or only very minor effect (Fig. 4).

In order to show the effects of the double mutant and notably the Lys192 mutant were not simply due to decreased activities of the enzymes, the chymotrypsin-like chromogenic substrate L-2130 (Suc-Leu-Leu-Val-Tyr-pNA, Bachem, Bubendorf, Switzerland) was used. No major differences in activities towards this substrate with any of the variants were seen (Fig. 4, right panel). These experiments were performed three times (fully independently) with almost identical results.

### Using structural information to evaluate the role of Arg143 and Lys192 in the interaction between the human chymase and the inhibitors

In order to determine the role of Arg143 and Lys192 in conferring the specificity in the interaction between the inhibitor and the enzyme, the available structural information on the HC and related enzymes were used to model their interactions.

**Figure 5:**
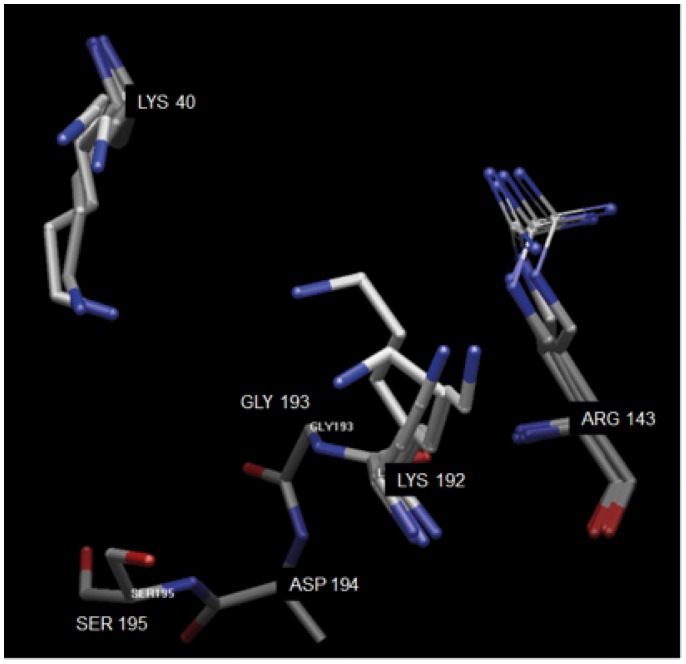
Visualization of the different positions of Lys40, Arg143 and Lys192 found in X-ray structures. The flexibility of the basic residues can be explained by crystal packing and the differences between the inhibitors used. X-rays structures included have the following PDB codes: 2HVX, 3S0N, 1T31, 3N7O.

BODIL 0.8.1 [18] an alignment and visualisation tool, was used to compare existing X-ray structures (PDB-codes: 2HVX, 3SON, 1T31, 3N7O; (http://www.rcsb.org/pdb/home/home.do; RSCB Protein Data Bank). The images clearly show the flexibility of Arg143 and Lys192 residues, due to different ligands and crystal packing depicted in Fig. 5. For a more detailed analysis of the protein structures 2HVX (structure of compound C) and an analogue of compound B, a benzimidazolone derivative (PDB: 3SON), have been used.

The X-ray structures of 2HVX indicates the phosphoric acid of compound C is serving as the ligand for the oxyanion hole as well as being used in the interaction to Lys192 (distances 2.9, 2.6 and 2.6 Å). Due to the strong binding in the oxyanion hole, the importance of Lys192 interaction should be minimal where Lys40 should have a better chance to replace this interaction. Gly193 & Ser195 of the serine protease form an oxyanion hole, a pocket, with a function in stabilizing a deprotonated oxygen in the tertrahedral reaction intermediates during the catalysis.

**Figure 6:**
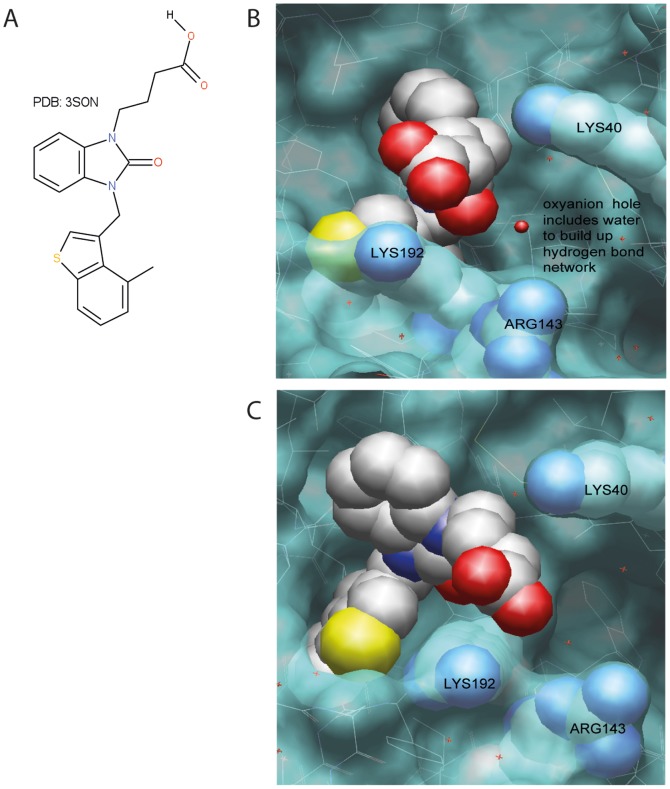
Structural support for the dependence of Arg143 and Lys192 in the interaction between a selected inhibitor (PDB:3S0N) and the HC. The X-ray structures of 3S0N were used to demonstrate the different interactions of Arg143 & Lys192 with the acidic side chain of the ligand. A, shows the ligand 4-{3-[(4-methyl-1-benzothiophen-3-yl)methyl]- 2-oxo-2,3-dihydro-1H-benzimidazol-1-yl}butanoic acid itself. B and C show different views of the interaction side depicted as Van der Waals surface forces of the protein in blue, and the ligand as well as the aa Lys40, Arg143 and Lys192 as a CPK model. In B, the oxyanion hole can be additionally seen including the crystal water building up a network with the imidazole carbonyl, and the aa Gly193 & Ser195 forming the oxyanion hole. The figure shows the distance between Lys192 and the acid side chain of the ligand even though large (>4 Å), is shorter compared to the distance with Arg143.

In 3S0N, the interaction with the oxyanion hole is mainly taken care of by the benzimidazolone itself. The interaction of the acid side chain is not well defined as distances are quite large (>4 Å) but Lys192 appears to be the most important one (Fig. 6). The analysis of the two X-ray structures suggest that acid chains attached to a tail like structure as in compound A, B and E, should be more strongly influenced by mutation of Lys192 & Arg143. The compounds in which the acid is more important for the oxyanion hole are less prone to being influenced by mutations in these two positions.

## Discussion

The HC is one of the human proteases that has attracted considerable interest from several of the major pharmaceutical companies as a potential medical target for anti-inflammatory treatments and also for the treatment of high blood pressure outside the renin-angiotensin system. Proteases constitute approximately 2% of the total human proteome [19] and an estimated 14% of all human proteases are being investigated as potential targets in drug development [20]. Experimental identification of the recognized substrate sequences can provide a strong basis for the design of potent inhibitors of these enzymes. Site-directed mutagenesis can then serve as a powerful tool in determining important interactions between the enzyme and substrate, which can facilitate the development process. A high specificity of the inhibitor is often essential to minimize the risk of unwanted side effects. Related enzymes can otherwise be affected by the inhibitor, which may result in effects of the inhibitor that are difficult to foresee and to control.

The observed preference for acidic aa in the P2'position of substrates for the HC may serve as a key characteristic to develop inhibitors that have a strong selectivity for this enzyme without affecting the activity of other related enzymes. Interestingly, the results from our analysis show that all five of the inhibitors tested were affected in their inhibitory ability by mutations in Arg143 and Lys192 of the HC. However, the effect was most pronounced for inhibitors A, C and E (Fig. 3). A synergistic effect of both residues was also observed for all five inhibitors (Fig. 3). The specificity was more dependent on Lys192 than Arg143. Therefore it appears as if the preference for negatively charged aa in the P2'position of the HC is a key characteristic in determining the specificity of these five inhibitors. In principal these experimental findings are supported by the analysis of the X-ray structures, where the efficiency of inhibition is not changed using compound C and D against the Lys192 or Arg143 single mutants. However, the double mutant has a relatively strong effect on the inhibition, which can possibly be explained by backwards folding of the basic amino acids Arg143 and Lys192 to increase the overall binding strength. In the case of compounds A and E, we see a strong influence by mutation in Lys192, which is also supported by the X-ray structure of the HC in combination with the inhibitor 3S0N. Compound B also shows a similar pattern, but with only a very minor effect of Arg143. Overall the experimental data provides good evidence that interaction with Lys192 is of importance and can be targeted for alternative design of inhibitors.

As mentioned above, a strong preference for acidic aa in the P2'position of substrates has been observed for the HC. Interestingly, at least three other chymases have a HC-like preference for acidic residues in the P2', namely the opossum α-chymase, rat mast cell protease (rMCP)-5 and mouse (m) MCP-4 [14], [21], [22]. All of these chymases contain Arg143 and Lys192 residues. The α-chymases from the macaque, baboon and guinea pig, the sheep mast cell protease, mMCP-5 and hamster chymase-2 are other chymases that also have Arg143 and Lys192 [9], [23]. Furthermore, the β-chymases rMCP-3, hamster chymase-1 and gerbil chymase-1 also hold Arg143 and Lys192. The guinea pig α-chymase was recently cloned and also shown to have Arg143 and Lys192 [23]. The preference for acidic P2' residues appears to be highly conserved among the α-chymases. However, there are exceptions: The gerbil is the only species of the previously mentioned that does not have an α-chymase with a predicted acidic P2' specificity. The gerbil α-chymase (chymase 2) has Lys143 and Lys192 [24]. Instead, the β-chymase gerbil chymase-1 has a predicted P2' specificity for acidic residues. Interestingly, the dog α-chymase holds Lys143 and Lys192 residues, and according to our analysis this chymase only has a weak preference for acidic residues in P2' [25]. Apparently, a minor change from the positively charged Arg to a slightly smaller but still positively charged side chain of a Lys residue in position 143 is enough to partially affect the preference for acidic P2' residues. Based on these findings one would expect that the five inhibitors developed against the HC also should show specificity for the Arg143 and Lys192 containing enzymes of other species. However, this may not necessarily be the case as the interactions are very complex. Weaker interactions in other regions may affect the specificity of the inhibitor and the influence of negatively charged residues in positions 40, 143 and 192. For example, several inhibitors developed against the HC have been shown to have very low activity against a few of the above listed chymases that have Arg143 and Lys192, for example sheep MC protease II and III and hamster MC protease 1 and 2 [26]. Even the dog chymase showed a very low sensitivity to these inhibitors indicating they have a very narrow species range. The only chymase in that analysis, which had a similar sensitivity to the inhibitors (used here) as the HC was a chymase from another primate, the macaque [26].

One of the most well characterized potential substrates for the HC is Ang I. The HC has been shown to efficiently convert Ang I to Ang II (cleavage of the Phe8-His9 bond in Ang I), without further degradation at the Tyr4-Ile5 bond. The structural requirements for this specificity have been addressed in several studies. Synergistic interactions of the P4 to P1 positions together with the dipeptidyl leaving group of Ang I was found to be important for efficient conversion by the HC [27], [28]. However, the side chains on the leaving group of Ang I do not seem to be important for the selectivity of the HC to convert Ang I [28]. Instead, the Lys40 side chain of the HC probably interacts with the negatively charged C-terminal carboxyl group of Ang I to stabilize the substrate [29]. The Lys40 and Arg143 residues of the HC have previously been analyzed for their role in the selective Ang I conversion. In this study, Lys40 but not Arg143 was found to contribute to the high specificity of the HC to convert Ang I to Ang II. The mutants of the HC used in this analysis were Lys40Ala and Arg143Gln [30]. The Arg143Gln mutant was actually shown to be more active than the wt in converting Ang I, indicating a minor role or no role at all of Arg143. The fact that mMCP-1, holding Lys143 and Met192 has a P2' specificity for Leu, is a good Ang I converter further indicated a lack of function for Arg143 and Lys192 in Ang I conversion [31]. The rat vascular chymase, with Arg143 and Thr192 residues thus not a predicted P2' specificity for acidic aa residues, also has very good Ang I conversion capability [32]. Interestingly, and in contrast to these predictions, we find that Lys192 but not Arg143 is of major importance for the cleavage rate of Ang I. Our results have previously clearly shown that both Arg143 and Lys192 residues of the HC are of major importance in mediating the specificity for acidic P2' side chains of substrates. The marked difference in the importance of Arg143 and Lys192 in determining substrate specificity between peptides and long substrates is striking. This clearly shows the importance of analyzing a broad range of different substrates when looking for the natural *in vivo* substrates. This finding also applies to the screening for potent chymase inhibitors, as these low molecular weight compounds may have different binding characteristics from larger natural protein substrates for the HC.

In conclusion, a more detailed knowledge of the specificity determining interactions may prove to be very valuable tools during the development of highly specific inhibitors of the HC and other medically important enzymes. The dependence of both Arg143 and Lys192 for the interaction with all five inhibitors tested in this study clearly points in this direction. A potentially very efficient way to obtain highly specific inhibitors could be to screen panels of inhibitors against both the wild-type and the mutant enzymes. This could highlight the maximum dependence of important residues in the specificity of the particular enzyme. Such inhibitors would have a good chance to primarily act on the enzyme of interest and leave related enzymes unaffected.
